# mRNA-LNP vaccines combined with tPA signal sequence elicit strong protective immunity against *Klebsiella pneumoniae*

**DOI:** 10.1128/msphere.00775-24

**Published:** 2024-12-31

**Authors:** Ting Huang, Siyou Che, Zheng Lv, Danrui Hao, Runyu Wang, Qinxuan Yi, Ling Mei, Yang Yuan, Hang Zou, Yidong Guo, Xinrong Wang, Yiwen Chu, Kelei Zhao

**Affiliations:** 1 Antibiotics Research and Re-evaluation Key Laboratory of Sichuan Province, School of Pharmacy, Chengdu University, Chengdu, China; 2 Engineering Research Center for Pharmaceuticals and Equipments of Sichuan Province, School of Pharmacy, Chengdu University, Chengdu, China; 3 Key Laboratory of Bio-resources and Eco-environment (Ministry of Education), College of Life Sciences, Sichuan University, Chengdu, China; University of Galway, Galway, Ireland, USA

**Keywords:** *Klebsiella pneumoniae*, mRNA vaccine, antibacterial immunity, virulence

## Abstract

*Klebsiella pneumoniae* is a prominent Gram-negative and encapsulated opportunistic pathogen that causes a multitude of infections such as severe respiratory and healthcare-associated infections. Despite the widespread anti-microbial resistance and the high mortality rate, currently, no clinically vaccine is approved for battling *K. pneumoniae*. To date, messenger RNA (mRNA) vaccine is one of the most advancing technologies and are extensively investigated for viral infection, while infrequently applied for prevention of bacterial infections. In the present study, we aim to construct a new mRNA vaccine encoding YidR or combining with a tissue plasminogen activator signal sequence for preventing *K. pneumoniae* infection. Adaptive immunity was determined in mRNA vaccines-immunized mice and the protective effects of mRNA vaccines were evaluated in *K. pneumoniae* infected models. The results showed that lipid nanoparticle (LNP)-YidR-mRNA vaccine was produced with good morphology, high the encapsulation efficiency, and the specific antigen was highly expressed in cells *in vitro*. In addition, immunization with either LNP-YidR or LNP-YidR-SP elicited a Th1-biased immune response, reduced bacterial load, and provided broad protection in the lung infection models. Importantly, the LNP-YidR-SP mRNA vaccine induced strong adaptive humoral and cellular immunity and increased the survivability of mice compared to the other groups. Our findings serve as a focal point for developing a potential mRNA vaccine against *K. pneumoniae*, indicating the potential of mRNA vaccines for improving next-generation bacterial vaccine.

**IMPORTANCE:**

*K. pneumoniae* is a notorious and clinical bacterium that is evolving in community-acquired and nosocomial settings. This opportunistic pathogen causes severe infectious diseases, including urinary tract infection and pneumonia, and causes a concerning global public burden. Despite efforts having been created to develop different types of *K. pneumoniae* vaccines, there is no licensed vaccine for preventing *K. pneumoniae* infection. Therefore, to develop an effective tactic is essential to combat *K. pneumoniae*-caused diseases. This study provides a novel vaccine strategy against *K. pneumoniae* and a potent platform to elicit high levels of humoral and cell-meditated immunity.

## INTRODUCTION

*Klebsiella pneumoniae* is a notorious and clinical bacterium that is evolving in community-acquired and nosocomial settings (1). This important opportunistic pathogen causes severe infectious diseases and causes a concerning global public burden (2). With the presence of versatile virulent genes, this bacterium is mainly categorized into hypervirulent *K. pneumoniae* and commonly classical *K. pneumoniae* (3, 4). Antibiotic therapy is a critical preference for treating severe infection caused by *K. pneumoniae*, while drug-resistant or multi-drug-resistant (MDR) strains pose a major challenge in clinical application (5).

Vaccines are a fundamental public health intervention and play an essential role in decreasing the burden of infectious diseases caused by *K. pneumoniae* (6). Current efforts have been created to develop different types of *K. pneumoniae* vaccines from traditional whole-cell vaccines to subunit vaccines (7). For example, whole-cell vaccines could prevent respiratory and urinary infections caused by *K. pneumoniae*, while many of these formulations displayed toxicity and had limited widespread use. Subunit *K. pneumoniae* vaccines have been shown to be a promising strategy to combat *K. pneumoniae* and have progressed to clinical trials including polysaccharide-based vaccines, protein-based vaccines, and conjugate vaccines. However, these vaccines also have their own limitations, such as low stability, low immunogenicity, and incomplete efficacy. Currently, there is no licensed vaccine for preventing *K. pneumoniae* infection (8). Hence, to develop an effective tactic is essential to combat *K. pneumoniae*-caused diseases. Nucleic acid vaccines based on messenger RNA (mRNA) were discovered decades ago with the aim of generating safe and effective vaccines, which are easier to produce (9). Compared to common vaccines, mRNA vaccines represent better alternative approaches for combating emerging infectious diseases (10). Unlike viral vaccines, mRNA may not integrate into the host genome or induce insertional mutagenesis (11). In addition, mRNA vaccines can be constructed in a cell-free manner at low cost and can effectively enhance host immune responses (12).

A recent research article indicated that a highly conserved virulent factor, called YidR, was identified in more than 300 different genomes from *K. pneumoniae* (13). YidR is a presumed ATP/GTP-binding factor which contributes to the hyperadherence phenotype of bacteria (14). Additionally, the YidR virulence factor could be a potential candidate for immunoprophylactic strategies against KP (15). Except for the antigen selection, the signal sequence also has great significance in the translation of targeted antigen and the improvement of mRNA-based vaccines against pathogens (16). Signal sequences (called signal peptides) are short N-terminal signals (normally 15–25 amino acids) which regulate the secretion and translocation of specific proteins in the cell (17). In an interesting study, the researchers demonstrated that the tissue plasminogen activator (tPA) signal sequence strongly drove a target protein into the cellular secretion pathway and enhanced the expression of target protein *in vivo* study (18). Moreover, it was noted that the signal sequences of DNA manipulated the expression and production of secretory proteins by an *in vitro* study (19). Nevertheless, whether an mRNA-based vaccine containing the YidR protein or tPA signal peptide can be used to control *K. pneumoniae*-related infections is still unclear.

In the current study, we attempted to construct new mRNA vaccines to treat *K. pneumoniae* infections. The program of vaccination includes an mRNA vaccine (lipid nanoparticle [LNP]-YidR) or combines tPA signal sequences (LNP-YidR-SP) as a molecular adjuvant to promote adaptive immunity in an animal model. The antigen-specific humoral or cellular immunity induced by the mRNA vaccines were determined. Moreover, those immunized mice were challenged with two different *K. pneumoniae* strains to evaluate the protective outcome of the designed vaccines. Consequently, this study provides the foundation for developing effective mRNA-based vaccines that elicit broad and complete protection in the fight against *K. pneumoniae* and its emerging MDR variants.

## RESULTS

### Preparation and characterization of LNP-YidR and LNP-YidR-SP mRNA vaccines *in vitro*

**Fig 1:**
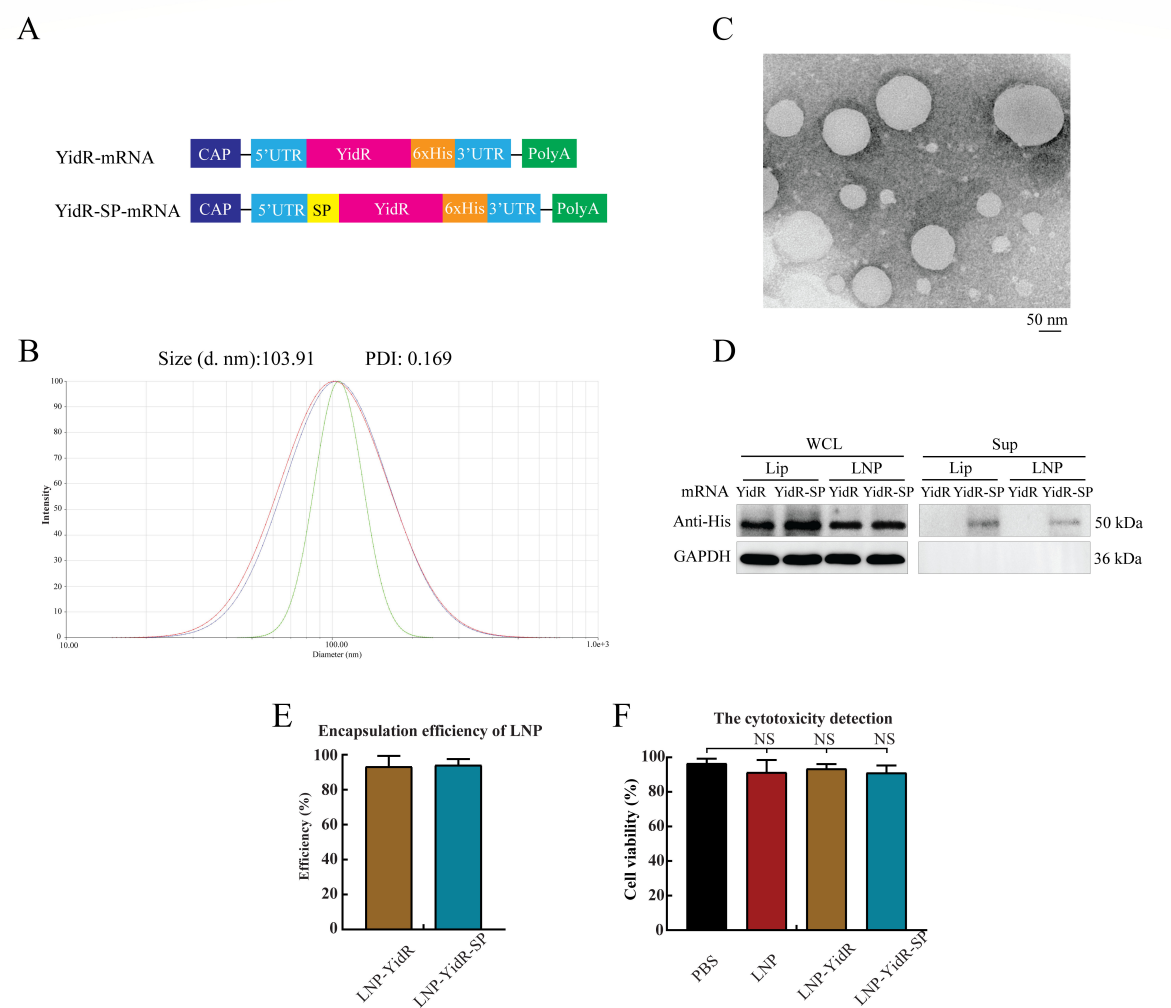
*In vitro* characterization of YidR-mRNA vaccine. (**A**) The schematic illustration of YidR-mRNA constructs. The mRNA constructs consist of 5′ cap followed by 5′-untranslated regions, with or without signal peptide (SP), YidR, 6xHis tag, 3′ untranslated region (UTR), and polyadenylation (polyA) tail. (**B**) The size distribution of LNPs was measured by a Malvern instrument. (**C**) The morphology of LNPs was detected by transmission electron microscopy. Scar bar, 50 nm. (**D**) The expression and secretion of YidR-mRNA vaccines was transfected into HEK-293T cells and detected by Western blotting. (**E**) The encapsulation efficiency of LNPs was determined by a Ribogreen assay. (**F**) The cytotoxicity of blank LNP and mRNA vaccines in HEK293 cells. Lip, lipofectamine 2000; LNP, lipid nanoparticle; NS, not significant; PDI, polydispersity index; PBS, phosphate-buffered saline; Sup, supernatant; WCL, whole-cell lysate.

To develop an effective and broad vaccine candidate against *K. pneumonia*, we designed two different mRNA vaccines encoding the YidR sequence (full length) with or without a signal peptide (Fig. 1A). LNP was prepared, and the average size of LNPs detected by a NanoBrook Omni instrument was 103.91 nm (Fig. 1B). Typical LNPs showed spherical and polydisperse characteristics as shown by the transmission electron microscope (TEM) method (Fig. 1C). Subsequently, purified mRNA was encapsulated into LNP via a cationic lipid-independent method to obtain LNP-YidR or LNP-YidR-SP for *in vivo* delivery. To confirm the expression of targeted YidR protein, LNP-YidR or LNP-YidR-SP was transfected into human embryonic kidney 293T (HEK293T) cells, and the level of protein was detected by Western blotting. As shown in Fig. 1D, the target proteins were highly expressed, and LNP-YidR-SP was secreted into the cell medium (Fig. S1). Furthermore, the encapsulation efficiency of the constructed mRNA vaccines was higher than 90% as analyzed by an RNA Quant Kit (Fig. 1E), and low cytotoxicity of mRNA vaccines was determined by using a Cell Counting Kit-8 (CCK-8) (Fig. 1F).

### Lethal dose determination of *K. pneumoniae* in mice

**Fig 2:**
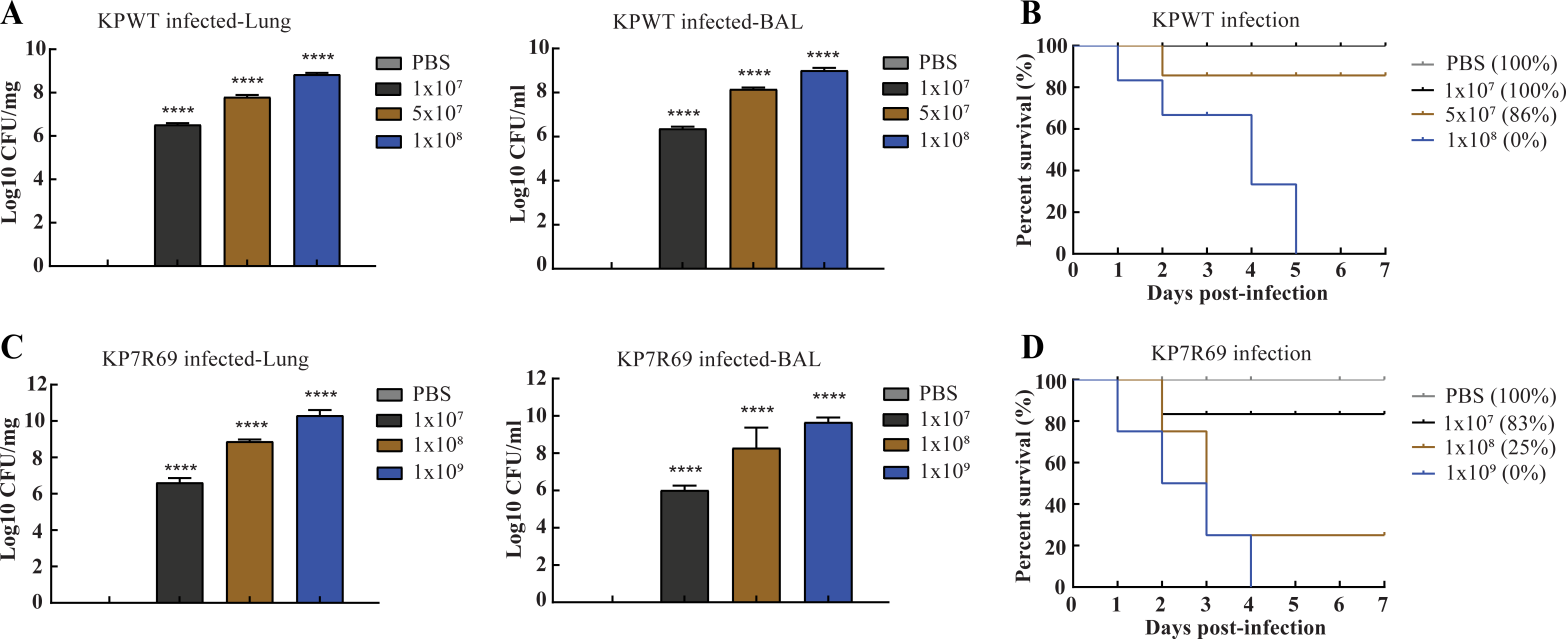
Determination of lethal dose using different loads of *K. pneumoniae* strains. (**A and C**) Bacterial burdens of tissues from different groups of infected mice were detected by a CFU assay. (**B and D**) Survival curves of infected mice were monitored for the subsequent 7 days. Data represent mean ± SEM. ****P < 0.0001.

To determine the lethal dose (LD100) of *K. pneumoniae* in a mouse model, the mice were intranasally infected with different bacterial loads of *Klebsiella pneumonia* wild-type (KPWT) or KP7R69 strain. The lungs or bronchoalveolar lavage fluid (BAL) fluids were isolated, and the bacterial counts of mouse lungs or BAL fluids are shown in Fig. 2A and C. As expected from the infection results, all mice infected with the 1 × 10^7^ CFU KPWT strain survived within 7 days, and all mice infected with 1 × 10^8^ CFU died at day 5 (Fig. 2B). Likewise, most mice infected with the 1 × 10^7^ CFU KP7R69 strain survived within 7 days, and all mice infected with 1 × 10^9^ CFU died at day 4 (Fig. 2D). Therefore, the lethal dose of *K. pneumoniae* in the respiratory infection model was identified as 1 × 10^8^ CFU KPWT or 1 × 10^9^ CFU KPR6 and was used for the downstream experiments. mRNA vaccines elevated antibody responses in mice.

**Fig 3:**
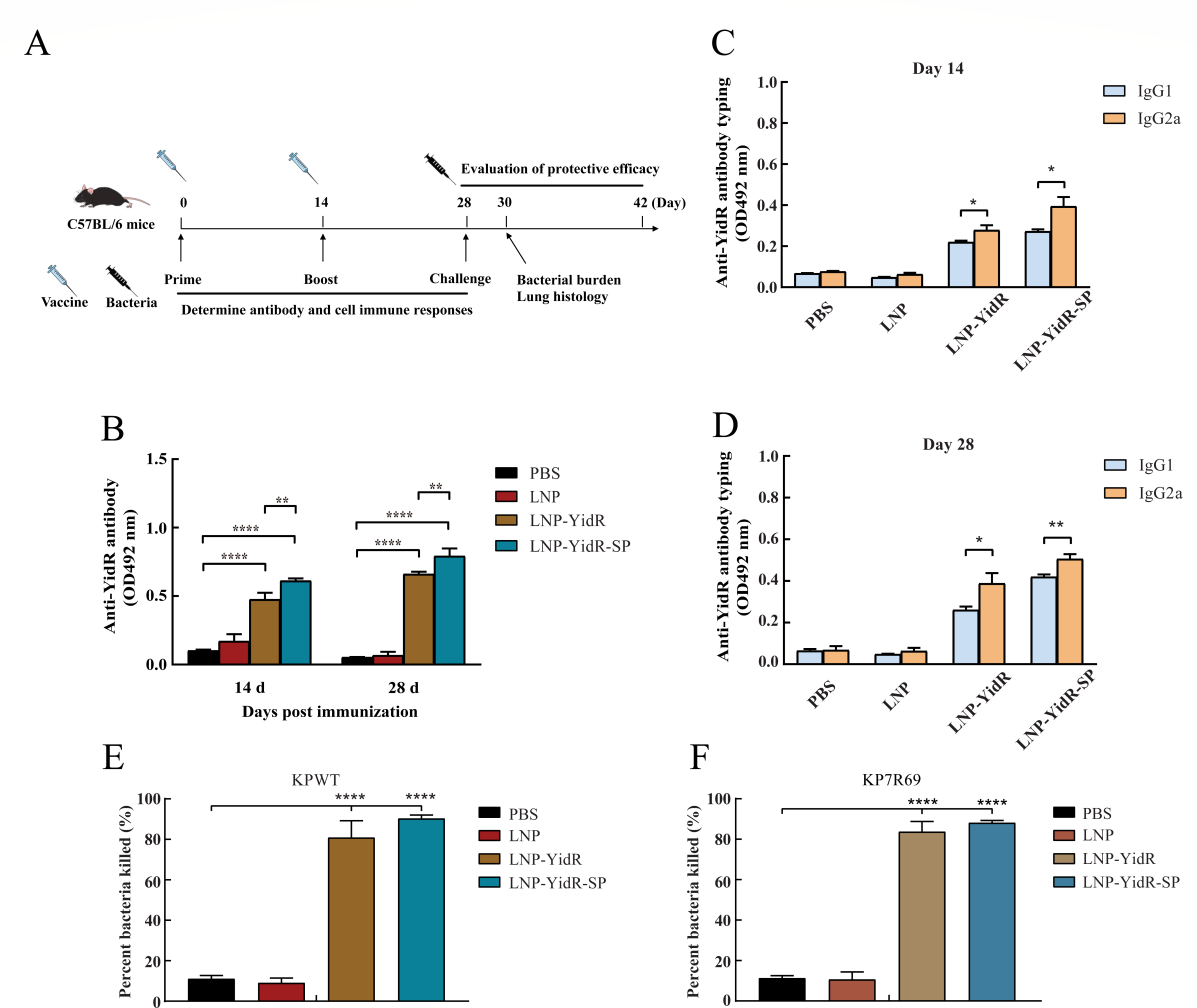
Antibody responses and opsonophagocytic killing activity of mice induced by the mRNA vaccines. (**A**) The experimental timeline was presented by a diagram; C57BL/6 mice were vaccinated or treated with PBS, followed by infection with *K. pneumoniae*. Vaccinations are indicated by the empty syringes; bacterial challenge is indicated by the black syringe. Mice were immunized with two mRNA vaccines and were boosted at day 14. (**B–D**) The anti-YidR IgG and IgG subtype (IgG1 and IgG2a) were detected at different time points by enzyme-linked immunosorbent assay. The OPK activity was detected in the serum of mice immunized with LNP-YidR and LNP-YidR-SP. (**E**) OPK activity against KPWT strain. (**F**) OPK activity against KP7R69 strain. Data represent mean ± SEM. *P < 0.05, **P < 0.01, ****P < 0.0001.

To investigate the immune response induced by LNP-YidR or LNP-YidR-SP, C57BL/6 mice were intramuscularly immunized twice with mRNA vaccines (Fig. 3A). As illustrated in Fig. 3B, immunization with the LNP-YidR elicited a high level of antibody production in mice at 14 days post-immunization (dpi), and the antibody was continuously increased at 28 dpi. In addition, antibody response of the LNP-YidR-SP group was greatly promoted compared with the LNP-YidR group and control group, while the antibody production was not substantially different in the control group. As indicated in Fig. 3C and D, the IgG subtype IgG1 and IgG2a were highly produced in the LNP-YidR and LNP-YidR-SP mRNA vaccine groups at 14 and at 28 dpi, while the production of IgG2a was greater than IgG1, revealing that immunization with both mRNA vaccines mainly promoted a Th1-biased immune response. To further investigate whether the sera of immunized mice could protect cells against KP infection, an OPK assay was performed to evaluate the bacterial killing activity of the specific antibodies by a complement-mediated pathway. As shown in Fig. 3E and F, bacterial killing activity was not detected in the sera samples from the phosphate-buffered saline (PBS) or LNP control group. In contrast, a highly bacterial killing activity was detected in mice immunized with mRNA vaccines (Fig. 3E and F).

### mRNA vaccines elicited strong cellular immune responses in mice

**Fig 4:**
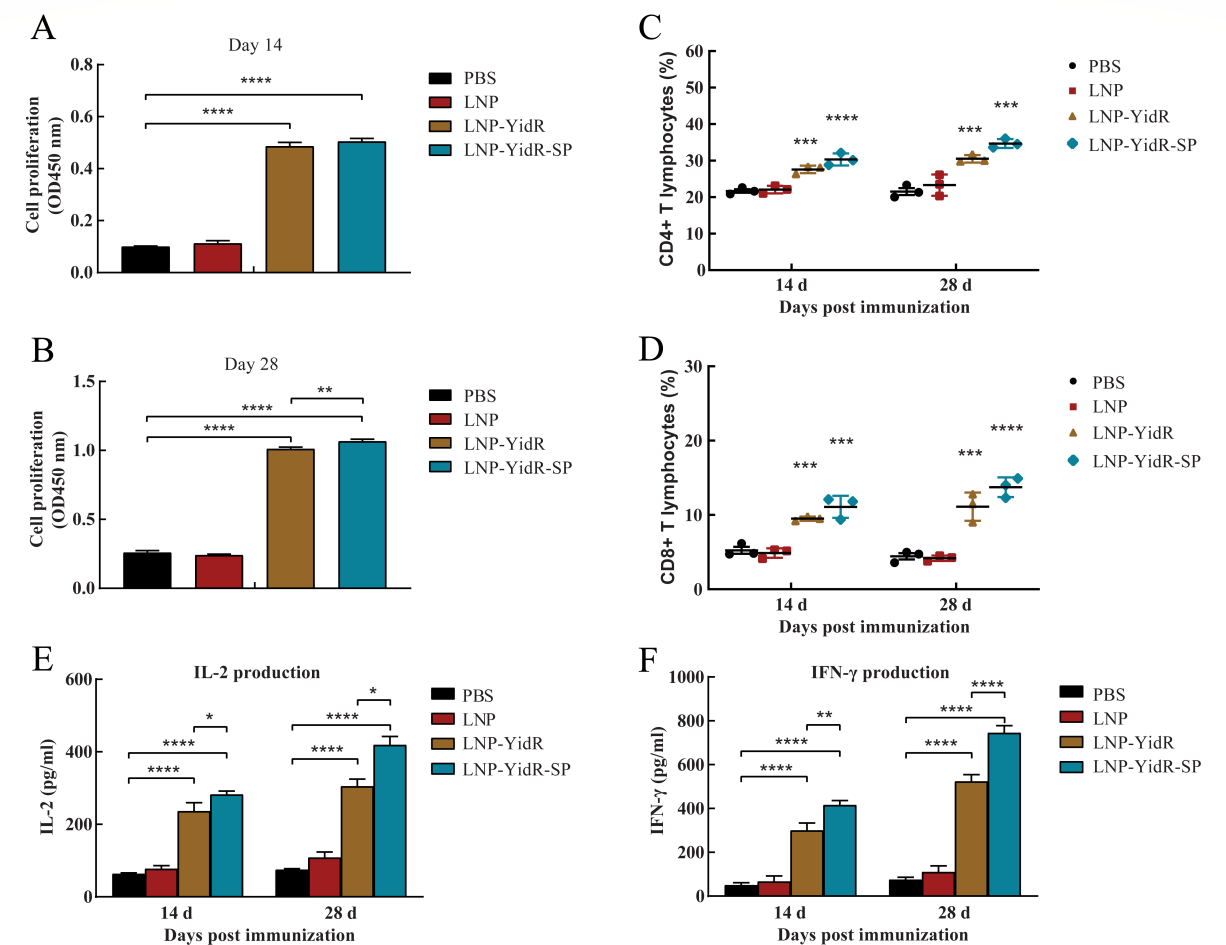
Cellular immune responses of mice activated by immunization with mRNA vaccines. (**A and B**) The spleen lymphocytes of immunized mice were separated as described in Materials and Methods, and the proliferation of spleen lymphocytes was detected by CCK-8 assay. (**C and D**) The CD4+ and CD8+ T lymphocytes of immunized mice were analyzed by flow cytometry at 14 and 28 days post-immunization. (**E and F**) The production levels of IL-2 (**E**) and IFN-γ (**F**) from the suspension of the spleen lymphocytes were assessed via an enzyme-linked immunosorbent assay kit. Data represent mean ± SEM. *P < 0.05, **P < 0.01, ***P < 0.001, ****P < 0.0001.

In addition to antibody responses, the antigen-specific cellular-mediated responses induced by the mRNA vaccines were evaluated. We found that the total spleen lymphocytes from the mice immunized with LNP-YidR significantly proliferated at 14 dpi by CCK-8 assay (Fig. 4A). Remarkably, the lymphocytes of the LNP-YidR group continually increased at 28 dpi after a booster immunization, and the LNP-YidR-SP group elicited much more lymphocytes than other groups (Fig. 4B). This effect was not detected in the LNP group and the PBS group (Fig. 4A and B). In addition, the CD4+ and CD8+ T lymphocytes from the mRNA vaccine group sustained a steady rise at 14 and 28 dpi compared to the PBS group (Fig. 4C and D). Larger numbers of T lymphocytes were detected in the mice of the LNP-YidR-SP group than the other groups. In a splenic cytokine assay, we found that mice immunized with LNP-YidR-mRNA vaccines showed high levels of interleukin (IL)-2 and interferon gamma (IFN-γ) production in the spleen lymphocytes between 14 dpi and 28 dpi (Fig. 4E and F). In contrast, no obvious change from the blank LNP or PBS group was observed (Fig. 4E and F). Notably, higher production of IL-2 and IFN-γ was detected in the mice immunized with LNP-YidR-SP at 14 and 28 dpi in comparison to the control group as shown in Fig. 4E and F. Together, our data demonstrate that encoding YidR in mRNA vaccine formulations elicited strong cellular immune responses in mice.

### Vaccination with LNP-YidR and LNP-YidR-SP protected mice from *K. pneumonia* infection

**Fig 5:**
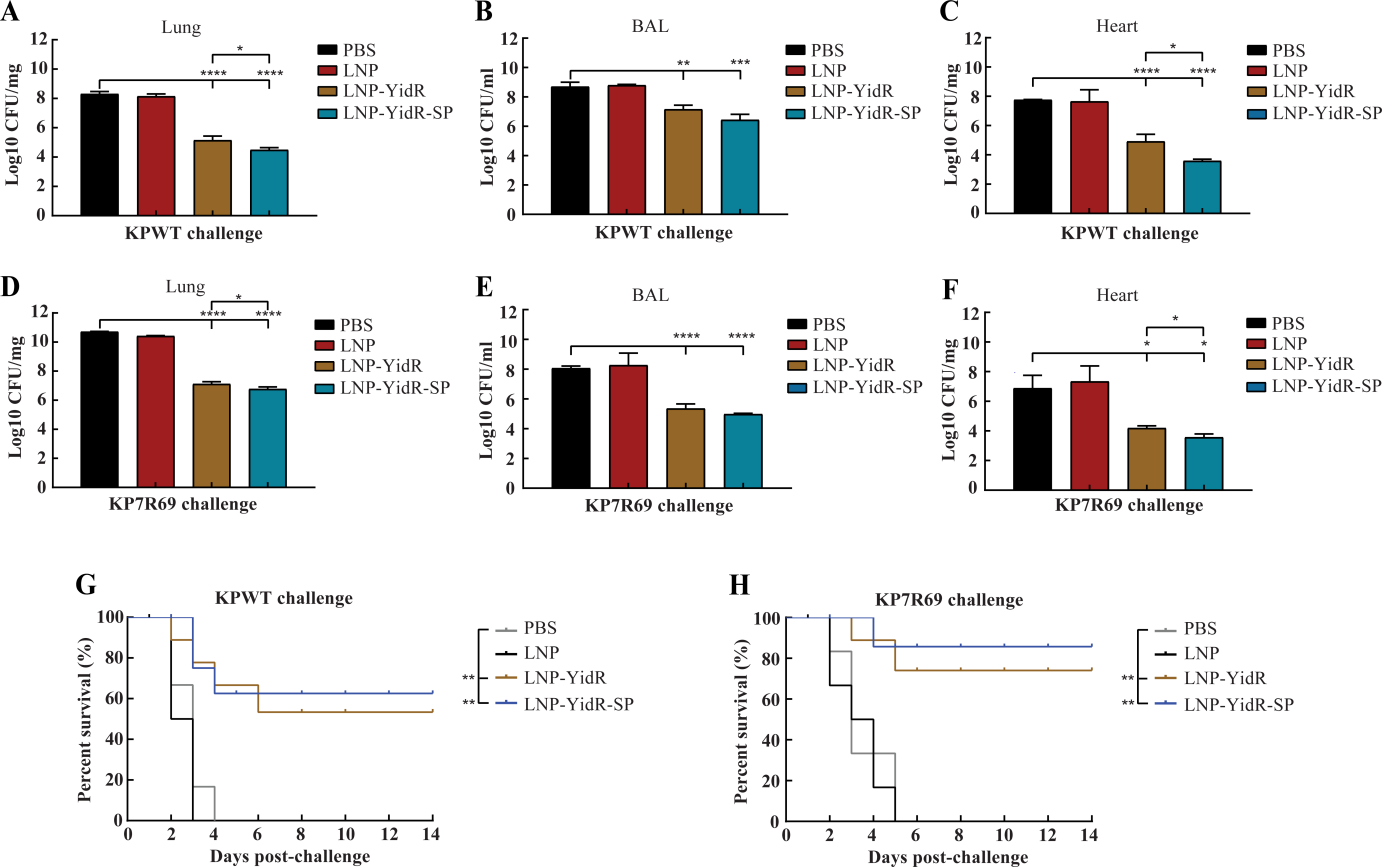
Bacterial burden and survivability of mRNA-immunized mice challenged with *K. pneumoniae*. (**A–H**) The immunized mice were infected intranasally at day 28 post-immunization with KPWT or KP7R69 strains. The bacterial loads of lungs (**A and D**), BAL (**B and E**), and hearts (**C and F**) were detected by CFU assay. Survivability of mRNA-immunized mice was monitored for 2 weeks (**G and H**). Survival curves of the immunized mice were assessed by log-rank (Mantel-Cox) test. Data represent mean ± SEM. *P < 0.05, **P < 0.01, ***P < 0.001, ****P < 0.0001.

To further estimate the broad protective efficacy of mRNA vaccines, we employed two different *K. pneumonia* strains (KPWT and KP7R69) for challenge experiments. The bacterial loads of KPWT in the lung tissues (Fig. 5A), BAL fluids (Fig. 5B), and heart tissues (Fig. 5C) of mRNA-immunized mice were lower than those of the control group. Consistently, the immunized mice of the mRNA vaccine group displayed less KP7R69 loads in the lung tissues (Fig. 5D), BAL fluids (Fig. 5E), and heart tissues (Fig. 5F) compared with the control group. Intriguingly, the group of LNP-YidR-SP resulted in a dramatic bacterial clearance of lung and heart tissues compared to the LNP-YidR group. For the survival analysis of challenged mice, the results showed that the mice in the PBS and LNP groups died within 4 or 3 days after infection (Fig. 5G). Conversely, almost half of the mice survived in the mRNA vaccine groups at 14 days via a KPWT challenge model (Fig. 5G). Likewise, more than 70% of mice immunized with LNP-YidR-mRNA vaccines survived during the KP7R69 challenge (Fig. 5H). Notably, most mice immunized with LNP-YidR-SP showed superior protection compared with the other groups.

**Fig 6:**
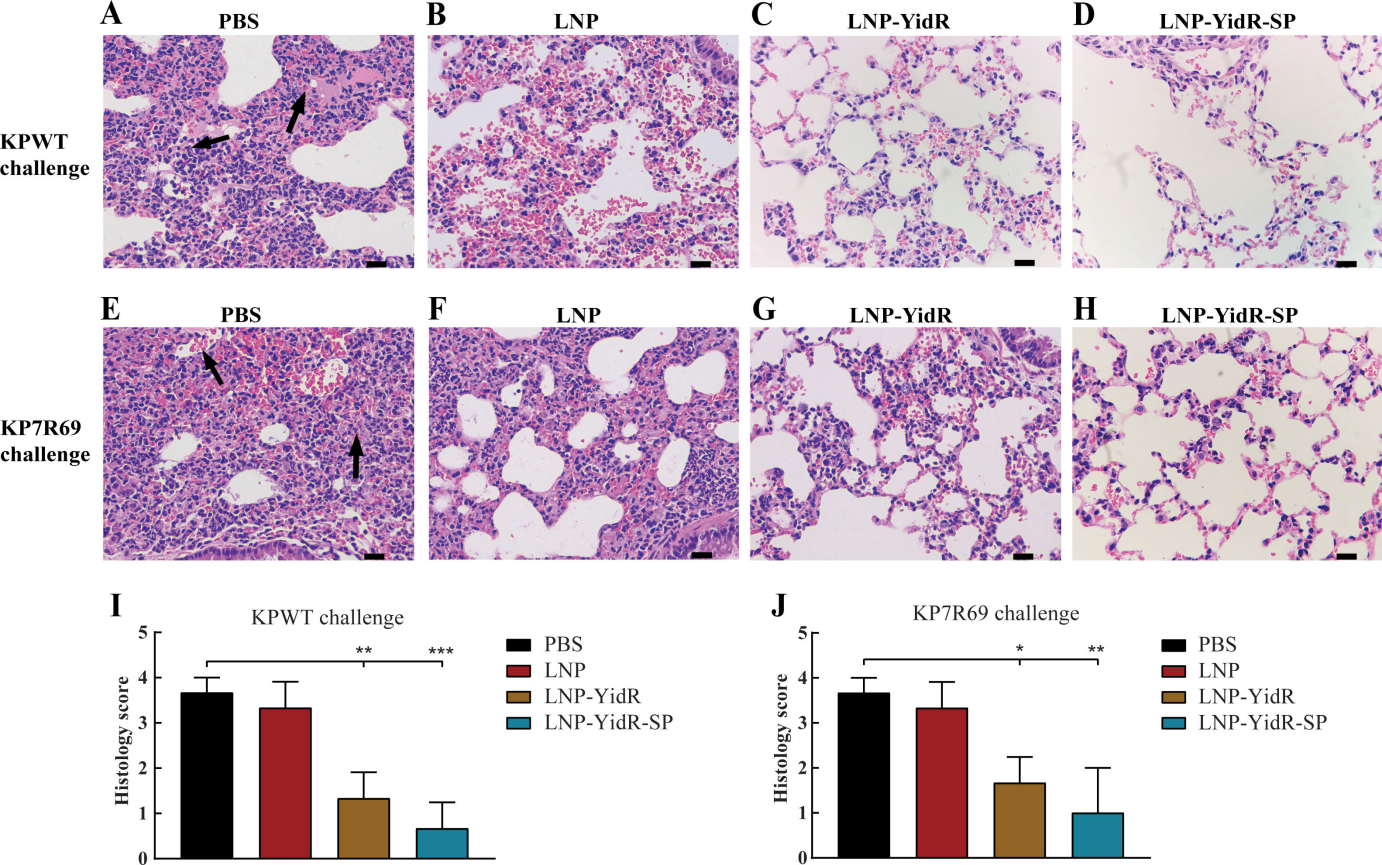
Histopathologic analysis of lungs in the mRNA-immunized mice challenged with *K. pneumoniae*. (**A–H**) The mice were infected with KPWT strain (**A–D**) or KP7R69 strain (**E–H**) at day 28. The lungs of mRNA-immunized mice were obtained for histopathologic analysis. (**A and E**) The cellular debris and abnormal cells of lungs are presented by black arrows. (**I and J**) Histology score in the infected lungs. Scale bar, 20 µm. *P < 0.05, **P < 0.01, ***P < 0.001.

For the histological analysis, the lungs of mice were collected 2 days after challenging with KPWT or KP7R69 and were subjected to hematoxylin and eosin staining. Severe inflammatory cell infiltration, destruction of the alveolar structure, and cellular debris were detected in the PBS or LNP group (Fig. 6A and B). In contrast, immunization with mRNA vaccines (LNP-YidR or LNP-YidR-SP) reduced the tissue damage and inflammatory changes in mice lungs after KPWT infection (Fig. 6C and D). Likewise, less inflammation or injury was observed in the mRNA vaccine-immunized group (Fig. 6G and H) compared to the PBS or LNP group (Fig. 6E and F) after the KP7R69 challenge (Fig. 6I and J). Altogether, these results suggest that both mRNA vaccines provided broad protection against lethal *K. pneumonia* challenge in mice.

## DISCUSSION

Despite the tremendous contributions of conventional vaccines in combating pandemic diseases, many infectious pathogens, including *K. pneumonia*, still lack accessible and efficacious vaccines. As a notorious clinical pathogen, *K. pneumonia* is a frequent cause of anti-microbial-resistant opportunistic infections (20). The prevalent antibiotic resistance of *K. pneumoniae* restrains intervention strategies, and the diverse strain heterogeneity of *K. pneumoniae* impedes the development of universal vaccines (7). Hence, creation of effective vaccines is urgently needed to control *K. pneumoniae* infection. In the present study, we designed a new mRNA-based vaccine expressing the highly conserved antigen YidR, encapsulated in LNP (LNP-YidR), to treat *K. pneumoniae* infection. In order to improve the immunogenicity of the LNP-YidR vaccine, we constructed an LNP-YidR vaccine fusion with tPA signal sequence (LNP-YidR-SP). It was found that the mRNA vaccine LNP-YidR induced high level of antibody and T cell-mediated immunity and remarkably protected the immunized mice from *K. pneumoniae* challenge. Importantly, the mRNA vaccine LNP-YidR-SP provided a broadly protective effect to prevent *K. pneumoniae*-caused infection in mice.

The selection of an antigen candidate is one of the challenges in the development of KP vaccines. A previous study indicated that a highly conserved and virulent factor, termed YidR, mediated the hyperadherence phenotype of bacteria (14). In addition, the tPA signal sequence is effective in facilitating the transport of targeted protein and can augment the expression and secretion of specific antigens (18). Thus, the YidR antigen was selected as a vaccine candidate, and tPA signal sequence was selected as molecular adjuvant in this study. LNP is the well-known delivery system for mRNA vaccine application, such as severe acute respiratory syndrome coronavirus 2 mRNA vaccines (21). Subsequently, the antigen sequence of the *yidR* gene was translated into nucleoside-modified mRNA and was encapsulated in the optimized cationic LNPs, including the tPA signal sequence as a molecular adjuvant. Under optimal conditions, the LNP-YidR showed good dispersity and a smooth surface. In addition, the *in vitro* HEK293T cells transfected with the constructed LNP-YidR or LNP-YidR-SP demonstrated that the expression of specific antigens was detected, and high encapsulation efficiency and low cytotoxicity of mRNA vaccines were determined. Altogether, these results suggested that the LNP-YidR or LNP-YidR-SP was safe with good bioactivity.

As discussed in a recent article, the YidR-specific antibody induced by vaccines is critical for bacterial clearance and host recovery (15). Evaluations of the IgG antibody levels showed that immunization with the LNP-YidR stimulated high levels of antibody responses in the mice. Remarkably, the mice of the LNP-YidR-SP group elicited higher production of antibodies than the LNP-YidR group and the control group. Furthermore, we found that the LNP-YidR or LNP-YidR-SP significantly promoted the bacterial killing activity. A previous study has demonstrated that the tPA signal sequence enhanced the immunogenicity of vaccines against bacterial pathogen (18). Therefore, our findings further confirmed the adjuvant effect of tPA in mRNA vaccine-immunized mice. Except for the antibody responses, the cellular-mediated responses also are the paramount protective strategies of the host against *K. pneumoniae* (7). Our findings indicated that vaccination with LNP-YidR enhanced the proliferation of lymphocytes and induced cellular immunity, which was required for protection against *K. pneumoniae* (15). Given cytokines were the critical reflector of the cellular immunity regulation for the host against bacterial pathogen (22), we further detected the production of IFN-γ and IL-2. Immunization with LNP-YidR enhanced production of protective IFN-γ and IL-2 in immunized mice. Moreover, LNP-YidR-SP significantly increased the production of IFN-γ and IL-2. These crucial collaborations may contribute to the protection of the host from *K. pneumoniae*-related infections. Therefore, our study demonstrated that the LNP-YidR or LNP-YidR-SP enhanced adaptive cellular immunity of mice.

Following *K. pneumoniae* challenge in mice, the bacterial load was reduced in the lung and heart tissues of LNP-YidR immunized mice, while less protection was detected in the control group. Mice vaccinated with our optimized candidate LNP-YidR-SP contained less bacteria in the lung and heart tissues compared to LNP-YidR immunized mice, revealing that most of the *K. pneumoniae* were eliminated by the host immunity and thus elevated the survival rates of immunized mice. Notably, immunization with LNP-YidR protected mice from KPWT infection and carbapenem-resistant KP7R69 with less the tissue damage and inflammatory changes in the lungs of challenging mice. Altogether, our novel mRNA vaccines could effectively induce specific humoral and cellular responses, which broadly protected mice from *K. pneumoniae* challenges.

Consequently, we conclude that mRNA vaccine LNP-YidR or LNP-YidR-SP induced high levels of immune responses. In particular, the tPA signal sequence served as a molecular adjuvant in combination with the mRNA vaccine to improve adaptive immunity and protection against *K. pneumoniae* infections. These findings could speed up the development of next-generation vaccines against rising anti-microbial resistance levels of bacterial pathogens such as *K. pneumonia*. Importantly, this study provides a promising strategy for decreasing public health risks. We acknowledge that the lack of immune dosage investigation in mRNA vaccines is a limitation of this study. Therefore, experiments are planned to estimate the vaccines in an immune dosage-dependent model or to determine the protective efficacy of the different vaccination approaches in other animal infection models in future studies. Moreover, using multi-virulence factor-based mRNA vaccines may provide a better strategy to combat *K. pneumoniae*-caused diseases.

## MATERIALS AND METHODS

### Chemicals and reagents

Ionizable lipid (L319) was purchased from MedChemExpress company (New Jersey, USA). Cholesterol and lysogeny broth (LB) was purchased from Solarbio Life Science (Beijing, China). Auxiliary lipid 1,2-distearoyl-sn-glycero-3-phosphocholine (DSPC) and polyethylene glycol were purchased from AVT Pharmaceutical Tech Company (Shanghai, China). Dulbecco's Modified Eagle Medium (DMEM) was purchased from Gibco (Grand Island, NY, USA). Lipofectamine 2000 was purchased from Thermo Fisher Scientific (Carlsbad, CA, USA).

### Mice, bacterial strains, and cells

Female C57BL/6 mice (7 or 11 weeks old) were purchased from Dashuo Co. Ltd. (Chengdu, China). All the animals were maintained under a standard housing condition, for example, feeding with pellet diet and sterile water in the room at around 22°C with 12 h light/dark cycle. The KPWT strain was from our laboratory and was incubated in LB under 37°C conditions (23). The clinical strain, carbapenem-resistant *K. pneumonia* 7R69 (KP7R69), was kindly provided by Dr. Qianglin Zeng (The Affiliated Hospital of Chengdu University). HEK293T cells were routinely maintained in complete DMEM (DMEM with 10% fetal bovine serum) at 37°C with 5% CO_2_. The recombinant protein His-YidR was preserved in this laboratory (15).

### Design and construction of mRNA vaccines

The protein sequence of the *yidR* gene was retrieved from the National Center for Biotechnology Information (GenBank number: NZ_KN046818.1). The mRNA vaccines were designed as previously reported with minor modification (18, 24). Briefly, the mRNA constructs consisted of 5′ untranslated regions (UTRs) of human β globulin, with or without signal sequence of tPA, YidR sequence, 6xHis tag, 3′ UTRs of human β globulin, and the 3′ end containing a polyadenylation sequence. The gene fragments (1,482 bp) described above were synthesized by Tsingke Co., Ltd. (Chengdu, China) and cloned into the a pGEM4Z vector (Promega, Madison, USA) to generate recombinant plasmid pGEM4Z-YidR or pGEM4Z-YidR-SP. The plasmids were identified by restriction endonuclease digestion and sequencing.

### Plasmid template amplification and linearization

The pGEM4Z-YidR or pGEM4Z-YidR-SP plasmids were transformed to *Escherichia coli* DH5α cells by the Ca^2+^-mediated transformation method. Then, the amplified bacterial cells were cultured on sterile LB agar plates with ampicillin antibiotic (50 µg/mL) at 37°C overnight. The single colony was picked up and added to the LB medium containing ampicillin in a conical tube, shaken (250 r/min) at 37°C overnight. The recombinant plasmids were extracted by a plasmid DNA extraction kit (Foregene, Chengdu, China) according to the manufacturer’s instructions. For the linearization, the restriction enzyme *Eco*RI was used to cut the site of the amplified plasmid. The linearized plasmids were recovered by a PCR purification kit (Foregene) following the manufacturer’s instructions.

### *In vitro* transcription and purification of mRNA

The linearized plasmids were used as templates for the *in vitro* transcription (IVT) reaction using a HiScribe T7 High Yield RNA Synthesis Kit (NEB, USA) according to the manufacturer’s instructions. Briefly, the IVT reaction was performed in 20 µL reaction volume and was incubated at 37°C for 2 h. Rnase-Free DNaseI was used to remove the residual DNA. Finally, the transcript production was capped by a Vaccinia Capping System (NEB) and was purified by a Monarch RNA cleanup kit (NEB).

### Preparation of the mRNA-LNPs

The mRNA constructs were formulated in LNPs as previously described with minor modifications (25, 26). Briefly, the LNPs were prepared as follows: dissolving L319, DSPC, cholesterol, and polyethylene glycol in absolute ethanol (50.0:38.5:10.0:1.5 molar ratio) to form the organic phase. The LNPs were then filtered through a 0.22 µm sterile filter, and the filtration was repeated three times. The purified mRNA samples were diluted in sodium citrate buffer (50 mM, pH 4) to form an aqueous phase. The prepared aqueous solution was added to the lipid film with a 1:10 M ratio of mRNA to lipid materials and mixed for 5 min using a Vortex-Genie2 device to obtain the mRNA-LNP mixture (Scientific Industries, Bohemia, USA). The ethanol was then removed, and the external buffer was replaced with sterile PBS (10 mM, pH 7.2) by the dialysis method. Finally, the mRNA-LNPs were concentrated to 1 mg/mL for the downstream experiments.

### Characterization of the LNPs

The particle size and polymer dispersity index value of LNPs were tested by a NanoBrook Omni Analyzer (Brookhaven Instruments Corporation, Holtsville, USA). The morphological and surface characteristics of LNPs were examined by JEM-F200 TEM (JEOL, Japan) as previously described (27).

### Determination of mRNA vaccine expression *in vitro* by Western blotting

The mouse monoclonal antibody against His-tag and GAPDH was obtained from Solarbio Life Science. The mRNA-YidR and mRNA-YidR-SP (2 µg) were transfected to HEK293T cells by LNP or Lipofectamine 2000. The cell samples were lysed in radioimmunoprecipitation assay buffer and were separated by the SDS-PAGE. The targeted proteins were immobilized by transferring onto a nitrocellulose membrane and were determined by a primary antibody and a secondary antibody conjugated to horseradish peroxidase (Solarbio Life Science). Enhanced chemiluminescence reagents were used for exposure as previously described (28).

### Encapsulation efficiency of mRNA-LNPs

The encapsulation efficiency of mRNA-LNP formulations was determined by an RNA Quant Kit (Vazyme, Nanjing, China) according to the manufacturer’s instructions. The RNA Quant reagent is an ultra-sensitive fluorescent nucleic acid stain which can detect free RNA that is not encapsulated by the LNP. LNP-YidR or LNP-YidR-SP obtained above was treated with Triton X-100 (1%) to release the encapsulated RNA and to obtain the amount of total RNA. The amount of unencapsulated mRNAs is detected before demulsification. The encapsulation efficiency of mRNA was calculated following the manufacturer’s instructions.

### mRNA-LNPs cytotoxicity assay

The cytotoxicity of mRNA-LNPs was determined in HEK293T cells by a CCK-8 in accordance with the manufacturer’s instructions (Mei5 Biotechnology, Beijing, China). Briefly, HEK293T cells were added into a 96-well plate, and the mRNA-LNPs (10 µg) were added into the plate. After 24–48 h of culture, a total of 10 µL CCK-8 solution was added to the plate and incubated for 3–4 h. Absorbance (450 nm) of the cells was recorded via a microplate reader.

### Determination of absolute lethal dose (LD100) of *K. pneumoniae*

The female C57BL/6 mice (11 weeks old) were randomly divided into eight groups (six mice per group). The infection experiments were performed as described in a previous article (29). Briefly, the animals were anesthetized with ketamine solution (50 µg/mL) by intraperitoneal injection. Afterward, 1 × 10^7^, 5 × 10^7^, and 1 × 10^8^ CFU of KPWT strain or 1 × 10^7^, 1 × 10^8^, and 1 × 10^9^ CFU of KPR6 strain in 30 µL sterile saline were intranasally instilled into the mice lungs, respectively. A control group was treated with 30 µL sterile saline without bacterial cells. The clinical signs of mice were observed for up to 7 days, including posture, coat quality, and body weight. At day 2 post-infection, the mice were sacrificed, and the lungs and BAL were separated and weighed. The isolated lung samples were triturated and homogenized in sterile PBS solution. Bacterial CFUs were determined by serial dilution in LB agar plates incubated at 37°C overnight.

### Immunization experiments

The C57BL/6 mice were randomly divided into four different groups (19 mice per group) as follows: PBS group, LNP group, LNP-YidR group, and LNP-YidR-SP group. After anesthetizing with ketamine solution, the mice were respectively immunized with mRNA vaccines (10 µg/mouse), LNP solution, or PBS solution (50 µL) by intramuscular injection and were further immunized on day 14 after the first vaccination by the same dose and volume.

### IgG antibody production in serum

Blood samples were harvested from the tail vein of mice at regular time points after vaccination (days 14 and 28). Briefly, the mice were anesthetized intraperitoneally with ketamine solution; the needle was inserted into the lateral tail vein of mice. Approximately 100 µL of blood was collected by the syringe and was transferred into a sterile 1.5 mL centrifuge tube. The serum of blood samples was collected, and YidR-specific antibody was detected by an indirect enzyme-linked immunosorbent assay (ELISA) assay as previously described (30). Moreover, antibody typing of the serum samples (IgG1 and IgG2a) was further assessed at the same time points (days 14 and 28) by the ELISA assay. The opsonophagocytosis activity of sera in the immunized mice was determined as previously described with minor modification (31). Briefly, RAW264.7 cells were adjusted to 5 × 10^5^ cells/well in a complete DMEM medium and were mixed with rabbit complement at 37°C with 5% CO_2_. The serum samples of mice were heat-inactivated for 20 min at 56°C and were mixed with 5 × 10^4^ CFU *K*. *pneumonia* at 37°C for 40 min. Opsonized bacterial suspension was added to RAW264.7 cells and was incubated at 37°C for 45 min. The reactions lacking the complement or the serum were also set as a control group. Bacterial count was determined by serial dilution in LB agar plates incubated at 37°C for 24 h. Opsonophagocytic activity of immunized groups was evaluated by calculating the percentage of reduction of bacterial numbers compared to the control group.

### Splenic lymphocyte proliferation assay

Proliferation of the splenic lymphocytes was detected by a CCK-8. The CCK-8 solution contains a water-soluble tetrazolium salt WST-8. WST-8 is reduced by dehydrogenase activities in cells for producing the soluble orange-color formazan dye. The amount of the formazan dye, produced by the activities of dehydrogenases in cells, is used to determine the quantity of living cells. Briefly, the splenic lymphocytes from the immunized or controlled mice were isolated via a mouse lymphocyte separation medium kit (Solarbio). The separated spleen lymphocytes (2 × 10^5^ cells/well in 96-well plate) were seeded in each well with complete growth medium (100 µL) and were treated with the His-YidR antigen protein (10 µg/mL) (15). The DMEM medium (100 µL) was set as the negative control group. All the cells were cultivated at the same condition (37°C, 5% CO_2_) for 24–36 h. A total of 10 µL CCK-8 solution was added to the plate and was incubated at the same condition (37°C, 5% CO_2_) for 4 h. Absorbance measured at 450 nm was used to evaluate the proliferation of the splenic lymphocytes by a microplate reader.

### Flow cytometry immunophenotyping

The CD4+ T lymphocyte or CD8+ T lymphocyte subsets from the mice spleen were evaluated via flow cytometry as shown in our published study (32). Briefly, a lymphocyte separation medium kit was applied to isolate the spleen lymphocytes as mentioned above. The separated spleen lymphocytes (1 × 10^5^ cells/well) were seeded in a 96-well plate with complete growth medium and were treated with the purified His-YidR protein (10 µg/mL). These cells were cultivated at 37°C for 24–36 h. Subsequently, the cells were transferred to 1.5 mL sterile microcentrifuge tubes and were fixed with 2% formaldehyde solution. The separated lymphocytes were stained by isotype control antibody, phycoerythrin-conjugated anti-mouse CD8 antibody, and FITC-conjugated anti-mouse CD4 antibody (BioLegend, San Diego, CA, USA) at 4°C for 1 h. The percentages of cells were further tested by a BD CantoII flow cytometer (BD Biosciences, Franklin Lakes, NJ, USA).

### Cytokine detection

The splenic lymphocytes from the immunized or controlled mice were separated as described above. The production levels of IFN-γ and IL-2 in the supernatant of spleen lymphocytes were detected via mouse cytokine ELISA kits (Solarbio) in accordance with the manufacturer’s instructions.

### Challenge experiments

The challenge experiments were performed 14 days after the second immunization as shown in a published report (29). The immunized or controlled mice were anesthetized with ketamine solution (50 µg/mL) by intraperitoneal injection. A total of 1 × 10^8^ CFU of KPWT strain or 1 × 10^9^ CFU of KP7R69 strain in 40 µL sterile saline was intranasally instilled into the mice lungs, respectively. Survivability and clinical signs of the infected mice were evaluated for up to 14 days, including ambulation, posture, and coat quality.

### Evaluation of bacterial load and histological analysis

At day 2 post-infection, the mice were sacrificed. The organs and BAL fluid were separated and weighed. The isolated heart and lung samples were triturated and homogenized in the sterile PBS solution. Triplicates were performed for the different groups (*n* = 3 mice per group). All the samples were serially diluted with sterile PBS. The bacterial loads in the different samples were detected on an LB agar plate as shown in a published article (28). Additionally, 4% paraformaldehyde solution was used to fix the separated lung tissues. Paraffin-embedded tissue sections were prepared and then were applied for hematoxylin-eosin staining as previously described (15).

### Statistical analysis

GraphPad Prism software (v.8.0.2) was applied for data and statistical analysis. Data were presented as mean ± SEM and were evaluated using one-way analysis of variance (Tukey’s test). *P* value thresholds corresponded to *P < 0.05.

## SUPPLEMENTAL MATERIAL

Fig. S1 msphere.00775-24-s0001.docx The full-length western blot images of Fig. 1D.

ASM does not own the copyrights to Supplemental Material that may be linked to, or accessed through, an article. The authors have granted ASM a non-exclusive, world-wide license to publish the Supplemental Material files. Please contact the corresponding author directly for reuse.
