# Supplementary material for: mRNA-LNP vaccines combined with tPA signal sequence elicit strong protective immunity against Klebsiella pneumoniae
Source: mSphere. 2024 Dec 31;10(1):e00775-24. doi: 10.1128/msphere.00775-24 (PMC11774038; doi:10.1128/msphere.00775-24)
Supplement: Fig. S1 — The full-length western blot images of Fig. 1D. [file msphere.00775-24-s0001.docx]

**

**

**Fig. S1** Full-length Western blot images. The full-length western blot image for Fig. 1D.
